# The COVID generation: Online dyslexia treatment equally effective as face-to-face treatment in a Dutch sample

**DOI:** 10.1007/s11881-023-00298-0

**Published:** 2024-01-11

**Authors:** Cara Verwimp, Anniek Vaessen, Patrick Snellings, Reinout W. Wiers, Jurgen Tijms

**Affiliations:** 1 Department of Developmental Psychology, University of Amsterdam, Amsterdam, The Netherlands; 2 Rudolf Berlin Center, Amsterdam, The Netherlands; 3 RID, Amsterdam, The Netherlands

**Keywords:** COVID-19, Dyslexia, Dyslexia treatment, Online intervention

## Abstract

Due to pandemic-induced lockdown(s) in 2020, dyslexia treatment was forced to move to online platforms. This study examined whether Dutch children who received online treatment progressed as much in their reading and spelling performance as children who received the usual face-to-face treatment. To this end, 254 children who received treatment-as-usual were compared to 162 children who received online treatment with Bayesian methods. The advantage of a Bayesian approach is that it can provide evidence for and against the null hypothesis whereas frequentist approaches only provide evidence against it. We found that children in the online treatment condition received slightly fewer treatment sessions but progressed equally after controlling for the number of sessions compared to the treatment-as-usual condition. These results have clinical and practical implications as they show that reading treatment can be successfully delivered online.

Developmental dyslexia, a neurodevelopmental disorder characterized by inaccurate and dysfluent reading, has prevalence rates of around 7% worldwide (Snowling, 2013). While significant progress has been made in remediating these severe reading difficulties, the pandemic-induced lockdown(s) in 2020 prompted a shift to online platforms. It is of great clinical interest to examine whether children were able to have their needs met under these circumstances, that is, whether children who received online treatment progressed as much on conventional reading and spelling measures as children who received the standard face-to-face treatment.

Dyslexia treatment commonly comprises systematic instruction of letter-speech sound correspondences and decoding strategies, along with applying these skills in reading and writing activities (Galuschka et al., 2014). These sessions are intense, systematic, and explicit, with an average length of 50 to 80 h (Torgesen, 2005). Previous studies showed significant improvement in Dutch reading programs for children with reading difficulties (e.g., Tijms, 2007, 2011; Tilanus et al., 2016). During this phonics-based program, children were provided with weekly treatment sessions at a clinical center in which intensive tutoring was followed by extensive practice. However, COVID-19 restrictions disrupted these one-to-one clinical sessions, leading professionals to shift diagnostic assessments, interventions, and supervision to online platforms.

Online videoconferencing for reading instruction is shown to be equally effective as face-to-face methods (Furlong et al., 2021). Dyslexia treatment, distinct from standard instruction, poses unique challenges as dyslexia frequently co-occurs with other developmental problems, including behavioral and affective problems (Margari et al., 2013), requiring specialized therapeutic skills for effective support. Scientific evidence on online provided dyslexia treatment is scarce, aside from a case study (Wright et al., 2011) and a pilot study (Kohnen et al., 2021) suggesting that online dyslexia treatment can be effective. Yet, small sample sizes and the lack of a face-to-face control group limits interpretation of these findings. Comparative, evidence-based findings are crucial to understand if online treatment meets dyslexic children’s needs and to examine groups at special risk.

This study used a clinical database of a nationwide, clinical center for learning disabilities in the Netherlands (Regional Institute for Dyslexia (RID)), to investigate if tele-practice for reading treatment is as effective as in-person therapy. We compared progress in online treatment during the pandemic with pre-pandemic treatment-as-usual data. Assessments were conducted at baseline (T0), after approximately 20 sessions (T1), and after approximately 40 sessions (T2), measuring gains in reading and spelling measures.

## Method

### Participants and procedure

Data was collected at RID over the period 2018–2021. All children were native Dutch speakers and attended regular elementary school (i.e., following the standard Dutch curriculum and not attending special education). Children had been referred to the center because of severe and persistent reading disabilities at school (i.e., below the 10th percentile on conventional reading measures or below the 10th percentile on spelling in combination with a score below the 16th percentile on reading) and resisted additional remedial support at school prior to referral. Employing an observational, pretest-treatment-posttest design, all children underwent diagnostic baseline assessment after which they obtained specialized reading treatment. Children in the face-to-face treatment condition (*n* = 254) finished their treatment before the first lockdown of the COVID-19 pandemic in The Netherlands (March 2020), whereas all children in the online condition (*n* = 162) started their first session around the first lockdown and followed the entire program online. Treatment progress was assessed halfway through the treatment period and at the end. This study obtained ethical approval from the ethics committee of the University of Amsterdam.

### Outcome measures

#### Reading

Participants’ ability to accurately and quickly decode words was measured with a Dutch word decoding test (DMT; Verhoeven, 1991). The test consisted of three different forms with each 150 words, divided over five columns. The orthographic structure difficulty increased across forms, with the first form consisting of monosyllabic VC, CV, and CVC words, the second form consisting of monosyllabic words with consonant clusters, and the third form consisting of polysyllabic words. All words were judged to be familiar to 6-year-old children (Schaerlaekens et al., 1999). For each form, children were instructed to read as many words as possible within 1 min. The total score was the sum of all correctly read words on the three forms.

#### Spelling

The ability to spell individual words was measured with a Dutch spelling test (PI dictee; Geelhoed & Reitsma, 1999). This task consisted of 135 items. For each item, a sentence was read aloud and the word that the child had to write down was repeated. Words increased in difficulty and syllabic complexity but were mainly consisting of only one morpheme. The total score was the number of correctly spelled words.

### Treatment

#### Face-to-face treatment

Following the dyslexia protocol in The Netherlands, children received a phonics-based, computer-aided treatment. The treatment consisted of approximately 40 sessions in which all Dutch letter-speech sound (L-SS) mappings were first explicitly taught by a therapist and consequently provided with high exposure by digital tools to obtain a transition from accurate, controlled to associative, automatic processing. Grapheme cards were used to explain and practice the speech sounds, and writing exercises took place on a whiteboard. Children received weekly, 50-min one-on-one sessions at the clinic and had to practice at home 5x/week for 15–20 min. All therapists were registered psychologists or speech therapists and were internally trained how to guide children through various types of exercises and constantly provided them with feedback.

Children were taught L-SS mappings step-by-step, starting with regular mappings followed by increasingly more complex, irregular mappings (i.e., short vowels, long vowels, diphthongs). After introducing the mapping, a touchscreen containing buttons corresponding to each Dutch speech sound was used. The touchscreen included icons to indicate the type of phoneme (e.g., ‘long vowel’), syllable icons (e.g., ‘stressed syllable’), and rule icons to perform certain operations (e.g., delete a selected grapheme). A Dutch word was aurally presented, and children were instructed to pronounce the presented vowels and identify the item by pushing the corresponding touchscreen buttons. Each button press produced a matching sound to direct attention to the matching of letters and speech sounds. Errors were corrected by the tutor and the computer screen.

Reading and spelling were also practiced in an explicit, structured manner. The focus during the treatment gradually shifted from easy word structures to complex words and loan words. An 80% accuracy rate was required before proceeding to the next phase with time-constraints adapted to children’s individual performance level.

#### Online treatment

**Fig. 1:**
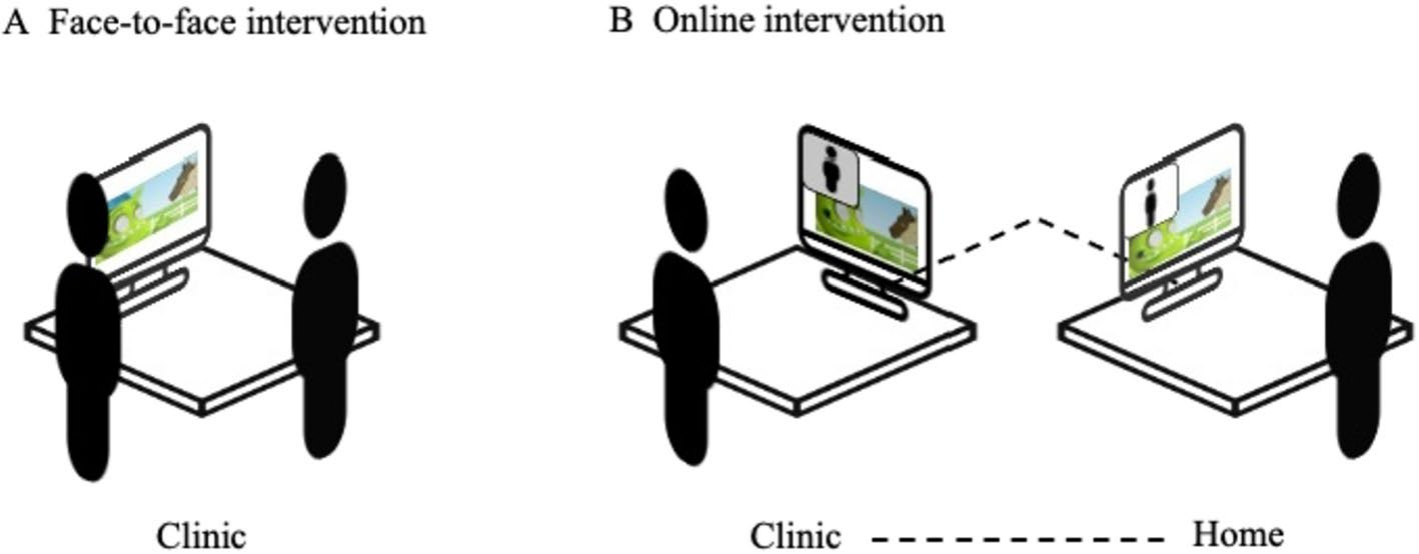
Intervention Set-Up

The online treatment comprised the same components, including the computer-aided software. Instead of weekly face-to-face sessions at the clinic, the weekly treatment sessions were provided via an online videoconferencing platform (Webex). During the online sessions, the therapist and child could communicate via a video-audio connection, while being able to practice using the computer-aided software (see Fig. 1). The session duration was identical to that of the face-to-face condition, as was the 5x/week practice sessions at home. Grapheme cards to explain and practice speech sounds were presented via online slides, and writing exercises took place on an online shared whiteboard.

### Data analysis

First, children with missing reading or spelling scores were removed from the current sample (*n* = 13). Baseline differences in age, intelligence, number of sessions, and baseline scores of word reading and spelling were examined with univariate Bayesian ANOVAs. Intelligence was measured with the WISC-III test battery (Kort et al., 2002). Additionally, we examined whether the two conditions differed in the compliance with homework assignments (% of homework sessions completed) as an index of treatment adherence. Second, to examine whether children who received online treatment improved equally compared to children who received face-to-face treatment, we performed a Bayesian ANCOVA with raw word reading and spelling post-treatment scores as dependent variables, treatment mode (face-to-face vs online) as a fixed factor and baseline word reading and spelling scores as covariates. Bayesian approaches have the advantage of estimating evidence for both the null and alternative hypothesis, whereas frequentist methods only provide evidence against the null hypothesis (Dienes, 2014). The Bayesian ANCOVA compared 4 models with varying predictors of the dependent variables: (1) a null model, (2) a model containing only treatment mode as a predictor, (3) a model containing only baseline scores as a predictor and (4) a model containing both treatment mode and baseline scores as predictors. We assessed model superiority using Bayesian Factors (BFs) with Jeffrey’s benchmarks for the interpretation of the strength of evidence; BFs between 1 and 3 were considered anecdotal, 3 to 10 as moderate, 10 to 30 as strong, 30 to 100 as very strong and > 100 as decisive evidence for a given model relative to another. Last, in order to examine whether children really improved their reading and spelling scores during treatment, standard reading and spelling scores of pre- and post-test were compared with a Bayesian paired sample T-test. Analyses were run in JASP using the default JASP priors (JASP team, 2022). Results were visualized in RStudio version 1.2.5033 (RStudio Team, 2022). Analyses were not pre-registered.

## Results

### Baseline measures

A total of 254 children received face-to-face treatment while 162 children received online treatment. Descriptives of the two groups are shown in Table 1. The Bayesian ANOVAs indicated that for age, the data were 4.74 times more likely to occur under the model including treatment compared to the null model, revealing a slight age difference at treatment initiation in the two conditions (face-to-face: *M* = 8.62 years, *SD* = 0.80; online: *M* = 8.85 years, *SD* = 0.85). In addition, face-to-face treatment involved slightly more sessions (*M* = 48.21, *SD* = 5.68) than online treatment (*M* = 44.15, *SD* = 5.41), with the data being more than 100 times more likely to occur under the model including treatment. This difference arose at random by municipality-level dyslexia care reimbursement differences. In the Netherlands, municipal resources affect the number of reimbursed sessions, and the number of children receiving either face-to-face or online treatment differed across municipalities. This yielded an at random difference in number of sessions attended in the face-to-face condition compared to the online condition. Therefore, the number of sessions was included as a covariate in the analyses. For intelligence and for reading scores, the data were approximately 8 times more likely to occur under the null model compared to the model including treatment and approximately 4 times more likely for spelling and phoneme awareness to occur under the null model. Last, for percentage of homework sessions, the data were 5 times more likely to occur under the null model. In other words, it was most probable that there were no differences between the two conditions in these baseline measures.

**Table 1:** Baseline Measures for Both Conditions Separately

	Face-to-face (*n* = 254)		Online (*n* = 162)	
	Mean	SD	Mean	SD
Age	8.62	0.80	8.85	0.85
N sessions	48.21	5.68	44.15	5.41
N reimbursed sessions	48.38	5.65	44.60	5.23
Intelligence	100.11	11.73	99.47	12.14
Homework sessions (%)	93.06	7.67	92.30	7.58
Reading (raw scores)	80.04	39.90	81.38	38.99
Spelling (raw scores)	38.43	18.54	40.83	20.72

N sessions = Number of sessions. % of homework sessions was used as an index of treatment adherence

### Treatment

**Fig. 2:**
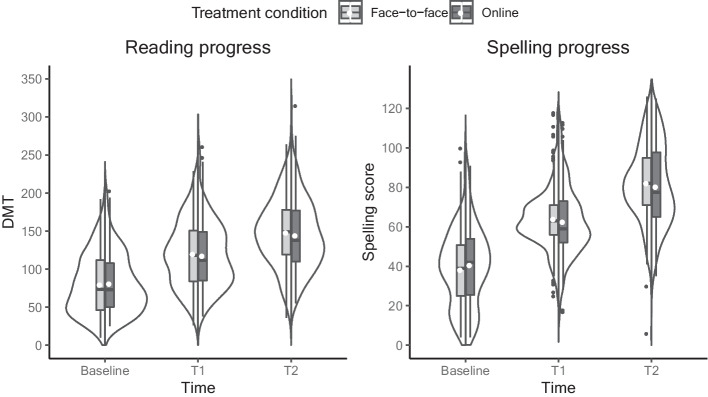
Intervention Outcomes for the Face-To-Face and Online Condition Separately. Note. Decoding ability (left) and spelling (right) progress for children who received the face-to-face treatment and the online treatment separately. The white dots represent the group means. DMT = Drie-minuten test (three-minutes test)

We used a Bayesian ANCOVA to compare reading and spelling outcomes for children who received face-to-face and online treatment. Intervention outcomes on both measures are depicted in Fig. 2. To control for sessions numbers, we compared eight models with varying predictors: (1) a null model, (2) a model only containing treatment mode, (3) a model only containing baseline scores, (4) a model only containing number of sessions, (5) a model containing baseline scores and number of sessions, (6) a model containing treatment mode and number of sessions, (7) a model containing baseline scores and treatment mode and (8) a model containing baseline scores, treatment mode and number of sessions.

For reading, posttest raw scores were greater than pretest scores in both conditions (face-to-face: pre: *M* = 80.04, *SD* = 39.90, post: *M* = 148.48, *SD* = 44.59; online: pre: *M* = 81.38, *SD* = 38.99, post: *M* = 144.69, *SD* = 47.39). Only model 5 had its model odds increased after observing the data (*BF*_*M*_ = 50.57). This means that the model including the baseline score and number of treatment sessions as predictors was the most probable (*P*(M|data = 0.88), with the observed data being 7.4 times more likely under this model than the model containing the baseline score, number of sessions and treatment mode. To account for model uncertainty, we performed Bayesian model averaging to the effects of all three predictors. The data was > 100 times more likely under models containing baseline scores and number of sessions as predictors of reading progress, but only 0.136 times as likely when including treatment mode. In sum, only baseline scores (mean effect = 0.95, 95% credible interval = [0.88,1.01]) and number of sessions (mean effect = 0.92, 95% credible interval = [0.46,1.38]) impacted reading scores after treatment and importantly, whether a child received face-to-face or online treatment had no effect.

For spelling, posttest raw scores were greater than pretest scores in both conditions (face-to-face: pre: *M* = 38.43, *SD* = 18.54, post: *M* = 82.34, *SD* = 19.40; online: pre: *M* = 40.83, *SD* = 20.72, post: *M* = 80.54, *SD* = 21.12). Model 5 (including baseline score and number of sessions), and model 7 (including baseline score, number of sessions and treatment mode) increased in model odds after observing the data (*BF*_*M*_ = 40.52 and *BF*_*M*_ = 1.21 for model 5 and 7 respectively). The model excluding treatment mode as a predictor was the most probable (*P*(M|data) = 0.85) with the observed data being 5.8 more likely under this model than the model containing all three predictors. To account for model uncertainty, we performed Bayesian model averaging to the effects of all three predictors. The data was > 100 times more likely under models containing baseline scores and number of sessions as predictor of spelling progress, but only 0.173 times as likely when including treatment mode. In sum, only baseline scores (mean effect = 0.78, 95% credible interval = [0.69,0.82]) and number of sessions (mean effect = 0.58, 95% credible interval = [0.34,0.81]) impacted spelling scores after treatment and importantly, whether a child received face-to-face or online treatment had no effect.

Finally, we performed Bayesian paired sample t-tests with age-normed T-scores (*M* = 50, *SD* = 10) to examine whether children improved their positions within the distribution of reading and spelling performance of an age-related normative sample. As the two conditions did not differ significantly, we pooled the data of the face-to-face treated and online group, revealing decisive evidence (BFs > 100) that posttest standard scores were greater than pretest scores for both reading (pre: *M* = 31.19, *SD* = 4.24; post: *M* = 33.84, *SD* = 6.78) and spelling scores (pre: *M* = 27.38, *SD* = 6.05; post: *M* = 36.85, *SD* = 10.17).

Overall improvement in reading scores post-intervention appeared lower than reported in previous studies (e.g., Tijms, 2007, 2011). One reason might be that in contrast to other reading tests using T-scores ranging from 20 to 80, the DMT has a lower bound of T = 27 and therefore is not able to detect changes below this boundary. Additionally, this discrepancy likely stems from averaging three DMT forms with increasing complexity. While most children improve in reading easy words, improvement might be less apparent in more complex polysyllabic words. We conducted a follow-up analysis of the three forms separately using categories based on the number of correctly read words compared to age-matched peers. These ranged from A to E, with A being the highest level and E being the lowest. Using a Bayesian Wilcoxon Signed-Rank Test, we found decisive evidence (BFs > 100) that children increased their reading performance on all three DMT forms post-intervention. Descriptive data indicated that 355 children performed in the lowest category for the first DMT form, 377 children performed in the lowest category for the second form, and 367 performed in the lowest category for the third form. Notably, 46%, 31% and 29% of children performed in at least one category higher after the intervention for the respective forms, indicating less pronounced improvement for the more difficult forms.

## Discussion

Due to the COVID-19 pandemic, many health services moved to online platforms. Although previous studies reported similar results using online literacy assessments compared to face-to-face assessments, results indicating the same for online-provided treatment is scarce. The current study used a clinical Dutch sample to evaluate whether children receiving online dyslexia treatment during the pandemic improved as much as those receiving traditional face-to-face treatment.

Comparing 254 children who received face-to-face treatment pre-lockdown to 162 children receiving all sessions online revealed no differences in reading and spelling progress after correcting for number of sessions. Importantly, the two conditions did not differ in treatment compliance assessed by completed homework assignments, which has been found to be an important predictor of intervention outcomes (Mausbach et al., 2010). These results suggest effective online delivery of reading treatment, carrying clinical and practical significance.

Tele-practice, proven effective in in speech- and language assessments (Taylor et al., 2014), also showed promise for dyslexia treatment (Kohnen et al., 2021; Wright et al., 2011). However, prior studies had limitations like small sample sizes and absence of a treatment-as-usual condition. Our results align with the notion that providing reading intervention via online platforms does not significantly impact intervention outcomes. While digital resource accessibility remains a challenge for some, tele-practice has promise for improved access to health services in rural areas or in special conditions such as pandemics. Related to this, access to services unavailable in some geographical regions is within one’s reach. Even when living abroad, individuals can obtain support in their native language. In addition, providing online treatment increases flexibility and reduces travel time. This provides flexibility in scheduling sessions for both clients and therapists, potentially shortening waiting lists. Financial resources can be attributed to providing treatment instead of paying overhead costs such as renting and maintaining office locations. Furlong and Serry (2022) found that even though many therapists did not provide tele-practice before the pandemic, most of them would continue delivering this service when all physical distancing regulations are abolished. Whether motivation in children receiving online treatment is equally high still needs to be examined in future studies, as well as whether some children benefit more from online treatment than others. Since dyslexia frequently co-occurs with other developmental problems, such as ADHD (Margari et al., 2013), some children probably benefit more from a face-to-face approach than online treatment.

There are some limitations in the current study that need to be considered. First, pandemic-induced lockdowns forced treatment to be online, preventing random allocation of children to the conditions and potentially may have biased the results. For example, the pandemic’s negative impact on children’s reading ability in general, especially in the youngest learners (Ludewig et al., 2022), could confound online intervention progress. A randomized controlled trial is needed for validation of our results. Second, the standard treatment in the Netherlands already includes digital tools, possibly minimizing differences between the online and face-to-face condition. Future studies should assess the generalizability of our findings to other treatment methods and languages.

In sum, this study supports existing literature on the equivalence of online and face-to-face dyslexia treatments in a Dutch clinical sample. Children showed equal reading and spelling improvement in both conditions. Future studies should explore individual variation in the effectiveness of online treatment and assess the generalization of our findings to other treatment methods and languages.

## Data Availability

Data can be made available upon reasonable request and after compliance with EC regulations.
